# Elevated tolerance of both short-term and continuous drought stress during reproductive stages by exogenous application of hydrogen peroxide on soybean

**DOI:** 10.1038/s41598-024-52838-2

**Published:** 2024-01-25

**Authors:** Oqba Basal, Tahoora Batool Zargar, Szilvia Veres

**Affiliations:** Department of Applied Plant Biology, Faculty of Agricultural and Food Sciences and Environmental Management, University of Debrecen, Debrecen, Hungary

**Keywords:** Plant sciences, Plant physiology

## Abstract

The global production of soybean, among other drought-susceptible crops, is reportedly affected by drought periods, putting more pressure on food production worldwide. Drought alters plants’ morphology, physiology and biochemistry. As a response to drought, reactive oxygen species (ROS) concentrations are elevated, causing cellular damage. However, lower concentrations of ROS were reported to have an alleviating role through up-regulating various defensive mechanisms on different levels in drought-stressed plants. This experiment was set up in a controlled environment to monitor the effects of exogenous spray of different (0, 1, 5 and 10 mM) concentrations of H_2_O_2_ on two soybean genotypes, i.e., Speeda (drought-tolerant), and Coraline (drought-susceptible) under severe drought stress conditions (induced by polyethylene glycol) during flowering stage. Furthermore, each treatment was further divided into two groups, the first group was kept under drought, whereas drought was terminated in the second group at the end of the flowering stage, and the plants were allowed to recover. After 3 days of application, drought stress significantly decreased chlorophyll-*a* and chlorophyll-*b*, total carotenoids, stomatal conductance, both optimal and actual photochemical efficiency of PSII (Fv/Fm and Df/Fm, respectively), relative water content, specific leaf area, shoot length and dry weight, and pod number and fresh weight, but significantly increased the leaf concentration of both proline and total soluble sugars, the root length, volume and dry weight of both genotypes. The foliar application of 1 mM and 5 mM H_2_O_2_ on Speeda and Coraline, respectively enhanced most of the decreased traits measurably, whereas the 10 mM concentration did not. The group of treatments where drought was maintained after flowering failed to produce pods, regardless of H_2_O_2_ application and concentration, and gradually deteriorated and died 16 and 19 days after drought application on Coraline and Speeda, respectively. Overall, Speeda showed better performance under drought conditions. Low concentrations of foliar H_2_O_2_ could help the experimented soybean genotypes better overcome the influence of severe drought during even sensitive stages, such as flowering. Furthermore, our findings suggest that chlorophyll fluorescence and the cellular content of proline and soluble sugars in the leaves can provide clear information on the influence of both drought imposition and H_2_O_2_ application on soybean plants.

## Introduction

Drought periods and intensities have reportedly increased during the past few decades, and are expected to further elevate along with globally rising temperature, with more devastating influence in the arid and semi-arid regions^1^. The increased global population is another factor imposing more pressure on both food production and water resources^2^. Drought stress is reported to be one of the most significant environmental factors affecting agricultural crop production and, hence, food security^3,4^. Decreased available water has serious consequences on the plants on several levels (morphological, physiological, molecular and biochemical), leading to hindered metabolic activities^5,6^.

To cope with drought conditions, plants undergo several changes on different levels, including reduced transpiration rate and stomatal conductance, decreased shoot growth and increased root growth^7–9^, elevated osmolyte production (e.g., soluble sugars, proline, etc.) which maintains cellular capacity of water retention through the osmolytes’ anti-dehydration characteristic^10^. Proline can also protect the enzymatic system during drought occasions with its protective ability for several enzymes, in addition to its role in redox regulation^11^. It was previously reported that proline accumulation under drought stress conditions in soybean was associated with better seed yield^12^. However, this defensive system can widely vary among plant species, and might differ depending on the species’ developmental stage.

In order for these changes to happen, chemical signals are initiated in the root system, including elevated abscisic acid levels, leading to reactive oxygen species (ROS) production in higher levels. If drought continues, excessive ROS production can lead to oxidative stress that can lead to massive damages on the cellular level^13^. It was reported earlier that ROS accumulation in the leaves can harm the photosynthetic pigments, leading to rapid leaf senescence^14,15^.

Low concentrations of ROS, however, were reported to potentially regulate the gene expression and the stress-responsive pathways, facilitate certain molecular and physiological alterations, cause a moderate accumulation of ROS which up-regulates the antioxidant system and, hence, partially alleviate the negative influence of several abiotic stresses, including drought^16–20^.

It was previously reported that the application of methyl viologent^21^, melatonin^22^, acetic acid^23,24^, abscisic acid^25^, salicylic acid^26,27^ and hydrogen peroxide^28^ positively helped in alleviating stress. Hydrogen peroxide is one of the most stable molecules among ROS that is naturally found in plant tissues, with several vital co-tasks on the cellular level including stomatal opening and cell growth and development^29–32^. The positive effects of exogenous H_2_O_2_ spray at different concentrations on several plant species are well documented; however, most of these studies focused on the seedlings of these species (e.g. 1.5 mM H_2_O_2_ on cucumber seedlings^33^, 0.5 mM H_2_O_2_ on tomato plants^34^, 1 MM H_2_O_2_ on soybean^35^, 10 mM H_2_O_2_ on maize seedlings^36^ or on the pre-treatment with H_2_O_2_ (e.g.^37^ on cucumber) rather than later developmental stages. Moreover, whether exogenous H_2_O_2_ spray can help the recovering plants after drought is finished and/or plants that are suffering from continuous drought is not well documented. It would therefore be of vital importance to address these questions properly.

Soybean is reported to be drought-susceptible crop^38^. Its susceptibility is widely different among its different varieties and, more importantly, depending on the developmental stage at which drought is imposed^39^. For example, it has been reported that drought during flowering stages^40^ and during the following stages^35^ massively reduces soybean yield by affecting both pod setting and seed filling^41^. Moreover, whether drought continues throughout several stages or is only occurring during certain developmental stage is another important issue to consider, and research is lacking on this particular issue. That said, it would be of considerable importance to understand the response of different soybean genotypes to either continuous or temporal drought at the more-sensitive reproductive stages. We hypothesized that low concentrations of H_2_O_2_ will have positive influence on the morpho-physiology and the biochemistry of soybean plants that suffer from drought stress during the sensitive flowering stage. We also hypothesized that the response of these drought-stressed plants to H_2_O_2_ would differ in case the drought was temporal as compared to continuous drought.This experiment aimed at evaluating the response of two soybean genotypes to short-term and continuous drought stress, in addition to evaluating the effects of exogenous application of H_2_O_2_ on the morpho-physiology and biochemistry of the drought-stressed soybeans.

## Materials and methods

This experiment was conducted through the hydroponic system in the controlled-climate chamber of the department of applied plant biology, University of Debrecen in 2022 to investigate the effects of exogenous application of different concentrations of hydrogen peroxide on soybean morpho-physiology and biochemistry under severe drought stress during the flowering (R1 and R2) stages^42^. In addition, this experiment aimed at monitoring the recovery path of soybean plants post-drought relative to continuous drought during reproductive stages (R1 onwards), and whether exogenously applied H_2_O_2_ might have a protective role. During the whole experimental period, the day/night temperature was kept at 26/19 °C with 65% relative humidity and light intensity of 300 µmol m^−2^ s^−1^ during the light period.

In a big field experiment, a total of 25 soybean genotypes were subjected to drought stress during 2017, 2018 and 2019 cropping years^43^. Based on their performance, two genotypes; Coraline (drought-susceptible) and Speeda (drought-tolerant) were chosen for this study. Severe drought stress was applied using polyethylene glycol (PEG 6000) (VWR International bvba Geldenaaksebaan, Leuven, Belgium) at a concentration of 10% (w/v) (equivalent to an osmotic potential of -0.19 MPa^44^) dissolved properly and completely in the nutrient solution of each pot (except control treatment). PEG is a widely used aqueous substance to conduct experiments on drought stress's effects on plants. It unites with water molecules, but can't enter the cells due to its high molecular weight. Drought stress was applied starting from R1 stage, and then either lifted at the end of R2 stage, or kept in place afterwards. Three H_2_O_2_ concentrations; 1, 5 and 10 mM were exogenously sprayed each other day throughout the flowering stages and a control treatment was alternatively sprayed with distilled water (DW). At the end of the flowering stage, the pots of each genotype were further divided into two groups; the first group was allowed to recover from drought stress by terminating PEG application, whereas the second group was kept under continuous drought stress conditions. Thus, there was 9 treatments for each genotype; 4 treatments sprayed with either 0, 1, 5 or 10 mM H_2_O_2_ (D, D1, D5 and D10, respectively) under drought stress imposed between R1 and R2 stages, 4 treatments sprayed with either 0, 1, 5 or 10 mM H_2_O_2_ (CD, CD1, CD5 and CD10, respectively) under continuous drought stress imposed from R1 stage onwards, in addition to a control treatment, where the plants were kept under optimum conditions and were sprayed with DW whenever the other treatments were sprayed with any concentration of H_2_O_2_.

To monitor the response of the different treatments, sampling for the different traits was made at 3 different occasions; 3 days after drought stress application (3 days after the beginning of R1 stage, equivalent to 51 and 59 days after sowing (DAS) in Coraline and Speeda, respectively), 3 days after terminating the drought stress as mentioned earlier (3 days after the ending of R2 stage and the beginning of R3 stage, equivalent to 64 and 73 DAS in Coraline and Speeda, respectively) and at the end of the experiment (at the end of R4 stage, equivalent to 89 and 101 DAS in Coraline and Speeda, respectively). At each sampling occasion, the second-most developed leaf was selected.The experiment was set in a randomized complete block design with 3 replications, so the final pot number was 54 (2 genotypes * 9 treatments * 3 replications).

Seeds of both genotypes were surface sterilized using 6% (v/v) H_2_O_2_ for 20 min, rinsed extensively with deionized water and germinated geotropically between moisten filter papers at 22 °C. After germination, 10 homogenous seedlings with good vigor were transferred into 3-L pots, and the number of seedlings was reduced later to 7 homogenous seedlings per pot. Each pot received 300 ml of dicot nutrient solution of the following: 0.7 mM K_2_SO_4_, 2.0 mM Ca(NO_3_)_2_, 0.1 mM KH_2_PO_4_, 0.5 mM MgSO_4_, 0.5 μM MnSO_4_, 0.1 mM KCl, 10 μM H_3_BO_3_, 0.2 μM CuSO_4_, 0.5 μM ZnSO_4_. Iron was supplied in the form of 10–4 M Fe-EDTA^45^, in addition to corresponding PEG solution. The nutrient solution was renewed every 3 days.

Stomatal conductance (gs) was measured with AP4 porometer (Delta-t devices, UK). Chlorophyll fluorescence was measured for dark-adapted leaves (20 min of dark adaptation) by attaching light exclusion clips to the central region of each leaf. Chlorophyll fluorescence parameters were measured with a portable chlorophyll fluorometer-*PAM-2100* (*WALZ,* Germany) as described by^46^. chlorophyll-*a*, chlorophyll-*b* and total carotenoids were calculated as described by^47^. The extract content of the pigment was measured with UV–VIS spectrophotometry (Metertech SP-830 PLUS, Taiwan) at three wavelengths; 480, 647 and 664 nm, and chlorophyll-*a* and chlorophyll-*b*, in addition to total carotenoid contents were determined according to^47^.

The specific leaf area (SLA) was measured as described by^48^.

Root and shoot dry weights were calculated after freeze-drying the samples (Christ Gefriertrocknungsanlagen Freeze Dryer, Type 101,041, Germany). Root and shoot lengths were measured using a standard ruler. Root volume was measured by placing the root in a suitable, graded tube containing a known volume of DW and then calculating the increase in the overall volume. The flower number was counted for each plant in each pot at R2 stage. Pod number and weight were calculated by harvesting the pods of 3 plants from each pot. Proline content was calculated as described by^49^. Total soluble sugar content was calculated as described by^50^.

GenStat 20th edition (VSN International Ltd, UK) software was used to conduct the Analysis of Variance test, followed by Duncan’s Multiple Range Test^51^ to identify the statistically different treatments. All values are the means of 3 replicates (indicated by columns within each figure) ± standard errors (indicated by vertical whiskers on each respective column).

### Plant material

The collection of plant material comply with relevant institutional, national, and international guidelines and legislation.

## Results

The group of treatments, where drought stress was continuously imposed starting from R1 stage (i.e., CD, CD1, CD5 and CD10), gradually deteriorated until complete death 16 and 19 days after drought stress application (i.e., 5 and 7 days after the beginning of R3 stage) on Coraline and Speeda, respectively.

### Root dry weight

**Figure 1:**
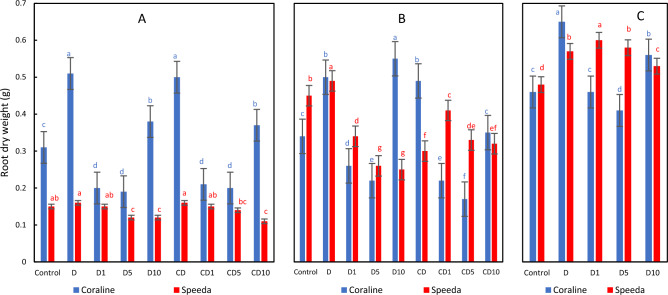
Root dry weight (g) of two soybean genotypes (Coraline and Speeda) at 3 different sampling dates (**A**: 3 days after drought stress application at R1 stage, **B**: 3 days after R3 stage started, **C**: at the end of R4 stage) as affected by hydrogen peroxide foliar spray application under drought stress conditions (**D**: drought from R1 till R2 stage, D1: drought from R1 till R2 stage + 1 mM hydrogen peroxide, D5: drought from R1 till R2 stage + 5 mM hydrogen peroxide, D10: drought from R1 till R2 stage + 10 mM hydrogen peroxide, CD: continuous drought starting from R1 stage, CD1: continuous drought starting from R1 stage + 1 mM hydrogen peroxide, CD5: continuous drought starting from R1 stage + 5 mM hydrogen peroxide, CD10: continuous drought starting from R1 stage + 10 mM hydrogen peroxide). All values are the means of 3 replicates (columns) ± standard errors (vertical whiskers). In each genotype, different letters indicate significant differences at .05 level as indicated by Duncan’s multiple range test.

The root dry weight of both genotypes was higher in treatments that were subjected to drought stress and received no foliar spray as compared to control treatments. The difference was more obvious and distinct in Coraline, where the root dry weight was even significantly higher (by 64.5%, 47.1% and 41.3% after 3 days of drought stress application, after 3 days from the beginning of R3 stage and at the end of R4 stage, respectively) than in the control treatment (Fig. 1). The application of H_2_O_2_ foliar spray decreased the dry weight of the roots of both genotypes as compared to the non-sprayed counterparts. However, the 10 mM concentration increased the root dry weight of Coraline as compared to both the 1 mM and the 5 mM concentrations but decreased it in Speeda. At podding stages, Coraline plants that were sprayed with 10 mM H_2_O_2_ had significantly higher root dry weight as compared to the 1 mM or 5 mM H_2_O_2_ concentrations; however, the non-sprayed plants still had significantly higher root dry weight. In Speeda, the 1 mM concentration resulted in significantly higher dry root weight as compared to the other concentrations. Similar results were recorded at the end of the podding stage of both genotypes (Fig. 1).

### Root length

**Figure 2:**
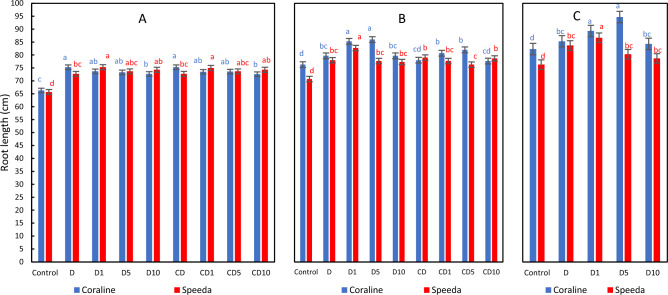
Root length (cm) of two soybean genotypes (Coraline and Speeda) at 3 different sampling dates (A: 3 days after drought stress application at R1 stage, **B**: 3 days after R3 stage started, **C**: at the end of R4 stage) as affected by hydrogen peroxide foliar spray application under drought stress conditions (**D**: drought from R1 till R2 stage, D1: drought from R1 till R2 stage + 1 mM hydrogen peroxide, D5: drought from R1 till R2 stage + 5 mM hydrogen peroxide, D10: drought from R1 till R2 stage + 10 mM hydrogen peroxide, CD: continuous drought starting from R1 stage, CD1: continuous drought starting from R1 stage + 1 mM hydrogen peroxide, CD5: continuous drought starting from R1 stage + 5 mM hydrogen peroxide, CD10: continuous drought starting from R1 stage + 10 mM hydrogen peroxide). All values are the means of 3 replicates (columns) ± standard errors (vertical whiskers). In each genotype, different letters indicate significant differences at .05 level as indicated by Duncan’s multiple range test.

After 3 days of drought stress application, the root length of both genotypes was significantly higher in all drought-stressed treatments than that of control treatment. In Coraline, the treatment which was sprayed with 10 mM H_2_O_2_ had significantly shorter roots (by 3.5%) compared to the treatment that was not sprayed. On the other hand, Speeda plants that received 1 mM H_2_O_2_ foliar spray had significantly longer roots (by 3.6%) compared to the non-sprayed counterpart. Within the group of treatments that was allowed to recover from drought, Coraline plants that were sprayed with either 1 mM or 5 mM H_2_O_2_ and Speeda plants that were sprayed with 1 mM H_2_O_2_ had significantly higher root lengths compared to their counterpart treatments that did not receive foliar spray. Within the group of treatments that were continuously under drought stress conditions, the root length of Coraline plants that were sprayed with either 1 mM or 5 mM H_2_O_2_ was significantly higher than the non-sprayed counterpart, whereas the foliar spray did not enhance this trait for Speeda plants at this point. At the end of the podding stage, the root length of all the treatments that received any concentration of H_2_O_2_ foliar spray was higher than the control treatments of both genotypes (Fig. 2).

After 3 days of drought stress application, the root length was not measurably different between the studied genotypes, regardless of H_2_O_2_ treatment and concentration. Later however, Coraline had longer roots in most treatments, especially within the group of treatments that were allowed to recover from drought. This last observation was more obvious at the end of the podding stage, where the root length of all treatments of Coraline was significantly higher than the counterpart treatments of Speeda (Fig. 2).

### Root volume

**Figure 3:**
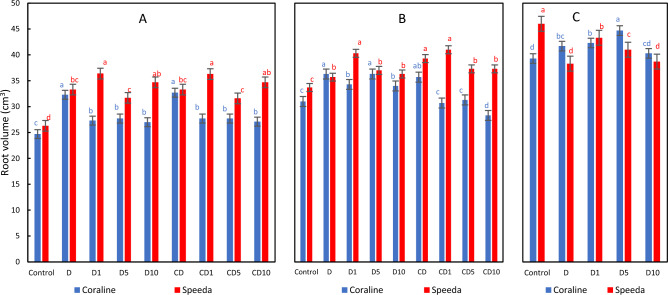
Root volume (cm^3^) of two soybean genotypes (Coraline and Speeda) at 3 different sampling dates (**A**: 3 days after drought stress application at R1 stage, **B**: 3 days after R3 stage started, **C**: at the end of R4 stage) as affected by hydrogen peroxide foliar spray application under drought stress conditions (**D**: drought from R1 till R2 stage, D1: drought from R1 till R2 stage + 1 mM hydrogen peroxide, D5: drought from R1 till R2 stage + 5 mM hydrogen peroxide, D10: drought from R1 till R2 stage + 10 mM hydrogen peroxide, CD: continuous drought starting from R1 stage, CD1: continuous drought starting from R1 stage + 1 mM hydrogen peroxide, CD5: continuous drought starting from R1 stage + 5 mM hydrogen peroxide, CD10: continuous drought starting from R1 stage + 10 mM hydrogen peroxide). All values are the means of 3 replicates (columns) ± standard errors (vertical whiskers). In each genotype, different letters indicate significant differences at .05 level as indicated by Duncan’s multiple range test.

Significant increase in the root volume of all sprayed plants from both genotypes was recorded 3 days after drought stress application as compared to control counterparts. In Coraline, H_2_O_2_ foliar spray application at any concentration significantly reduced the root volume as compared to the non-sprayed treatment (by 15.5%, 14.2% and 16.4% in the treatments that received 1, 5 and 10 mM, respectively), whereas a 1 mM H_2_O_2_ foliar spray significantly increased the root volume of Speeda plants. In Coraline, the root volume was significantly higher for the recovering plants that were sprayed with 5 mM H_2_O_2_ foliar spray compared to the other concentrations, whereas the foliar spray at all concentrations resulted in significant root volume decrease in the treatments that were continuously subjected to drought. In Speeda, the root volume of the treatment sprayed with 1 mM H_2_O_2_ was significantly higher than the other sprayed treatments for both groups (recovered, unrecovered). Similar conclusion was obtained on the recovered groups of both genotypes at the end of the podding stage (Fig. 3).

### Shoot dry weight

Drought stress significantly decreased the shoot dry weight of both genotypes 3 days after application, and the foliar spray of H_2_O_2_, regardless of concentration, did not enhance this trait. After removing drought, the shoot dry weight of the plants that were sprayed with any concentration of H_2_O_2_ foliar spray was significantly higher compared to the plants that were allowed to recover without receiving any concentration of foliar spray. Under continuous drought stress conditions, the shoot dry weight of both genotypes was significantly less than that of the recovering plants. However, the foliar spray of H_2_O_2_ at any concentration significantly enhanced the dry shoot weight in Coraline (by 13.6%, 22.4% and 17.7% in CD1, CD5 and CD10 treatments, respectively as compared to CD treatment), but not in Speeda. By the end of the podding stage, the effect of the foliar spray on the shoot dry weight was more measurable in Coraline than in Speeda.

**Figure 4:**
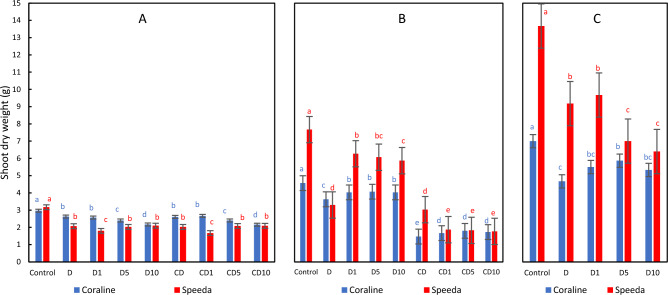
Shoot dry weight (g) of two soybean genotypes (Coraline and Speeda) at 3 different sampling dates (**A**: 3 days after drought stress application at R1 stage, **B**: 3 days after R3 stage started, **C**: at the end of R4 stage) as affected by hydrogen peroxide foliar spray application under drought stress conditions (**D**: drought from R1 till R2 stage, D1: drought from R1 till R2 stage + 1 mM hydrogen peroxide, D5: drought from R1 till R2 stage + 5 mM hydrogen peroxide, D10: drought from R1 till R2 stage + 10 mM hydrogen peroxide, CD: continuous drought starting from R1 stage, CD1: continuous drought starting from R1 stage + 1 mM hydrogen peroxide, CD5: continuous drought starting from R1 stage + 5 mM hydrogen peroxide, CD10: continuous drought starting from R1 stage + 10 mM hydrogen peroxide). All values are the means of 3 replicates (columns) ± standard errors (vertical whiskers). In each genotype, different letters indicate significant differences at .05 level as indicated by Duncan’s multiple range test.

After drought application, Coraline plants had higher shoot dry weight; however, Speeda plants showed measurably higher values in most treatments during the following stages (Fig. 4).

### Shoot length

**Figure 5:**
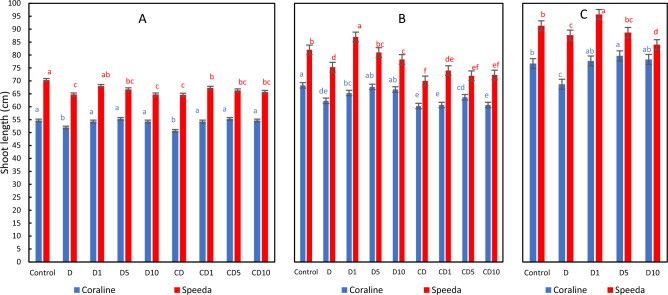
Shoot length (cm) of two soybean genotypes (Coraline and Speeda) at 3 different sampling dates (**A**: 3 days after drought stress application at R1 stage, **B**: 3 days after R3 stage started, **C**: at the end of R4 stage) as affected by hydrogen peroxide foliar spray application under drought stress conditions (**D**: drought from R1 till R2 stage, D1: drought from R1 till R2 stage + 1 mM hydrogen peroxide, D5: drought from R1 till R2 stage + 5 mM hydrogen peroxide, D10: drought from R1 till R2 stage + 10 mM hydrogen peroxide, CD: continuous drought starting from R1 stage, CD1: continuous drought starting from R1 stage + 1 mM hydrogen peroxide, CD5: continuous drought starting from R1 stage + 5 mM hydrogen peroxide, CD10: continuous drought starting from R1 stage + 10 mM hydrogen peroxide). All values are the means of 3 replicates (columns) ± standard errors (vertical whiskers). In each genotype, different letters indicate significant differences at .05 level as indicated by Duncan’s multiple range test.

The soot length of both genotypes significantly decreased as a result of drought stress application. However, the H_2_O_2_ foliar spray application, of any concentration in Coraline and of 1 mM in Speeda, could significantly enhance this trait. When plants of both genotypes were relieved from drought and allowed to recover, the groups of treatments that received any concentration of H_2_O_2_ foliar spray showed significantly higher shoot length compared to the non-sprayed counterparts. Under continuous drought conditions, only the concentrations of 5 mM and 1 mM H_2_O_2_ foliar spray could result in better shoot lengths in Coraline and Speeda, respectively. At the end of podding stage, all Coraline plants that received H_2_O_2_ foliar spray showed significantly better shoot length (better even than the control), whereas that positive effect was noticed only on the treatment of Speeda that received 1 mM H_2_O_2_ foliar spray, where the shoot length was 9.1% higher than that of the treatment that was not sprayed (D) and 4.8% higher than that of the control treatment (Fig. 5).

Regardless of drought and H_2_O_2_ application, the shoot length of Speeda was significantly higher than that of Coraline through the whole experimental period.

### Specific leaf area

**Figure 6:**
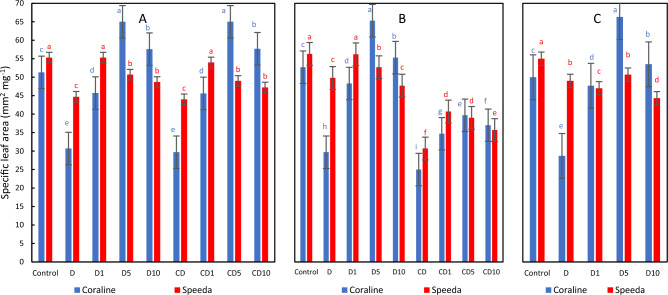
Specific leaf area (mm^2^ mg^-1^) of two soybean genotypes (Coraline and Speeda) at 3 different sampling dates (**A**: 3 days after drought stress application at R1 stage, **B**: 3 days after R3 stage started, **C**: at the end of R4 stage) as affected by hydrogen peroxide foliar spray application under drought stress conditions (**D**: drought from R1 till R2 stage, D1: drought from R1 till R2 stage + 1 mM hydrogen peroxide, D5: drought from R1 till R2 stage + 5 mM hydrogen peroxide, D10: drought from R1 till R2 stage + 10 mM hydrogen peroxide, CD: continuous drought starting from R1 stage, CD1: continuous drought starting from R1 stage + 1 mM hydrogen peroxide, CD5: continuous drought starting from R1 stage + 5 mM hydrogen peroxide, CD10: continuous drought starting from R1 stage + 10 mM hydrogen peroxide). All values are the means of 3 replicates (columns) ± standard errors (vertical whiskers). In each genotype, different letters indicate significant differences at .05 level as indicated by Duncan’s multiple range test.

Drought stress application resulted in significant reductions in the specific leaf area (SLA) of both genotypes as compared to control counterparts. However, the application of H_2_O_2_ foliar spray significantly increased SLA, regardless of its concentration. Compared to control plants, the application of 5 mM and 10 mM H_2_O_2_ foliar spray on Coraline resulted in significantly higher SLA (by 26.7% and 12.3%, respectively) after 3 days of drought application, whereas only the 1 mM H_2_O_2_ concentration could enhance this trait in Speeda. Under continuous drought stress conditions, the application of H_2_O_2_ foliar spray could significantly increase SLA in both genotypes as compared to the non-sprayed counterparts. Recovering Coraline plants sprayed with either 5 mM or 10 mM H_2_O_2_ could maintain higher SLA values as compared to control plants, whereas Speeda plants could not. Similar findings were observed at the end of the podding stge (Fig. 6).

At all sampling dates, SLA of Speeda was higher in control and drought-stressed treatments that did not receive any foliar spray; however, Coraline plants that were sprayed with either 5 mM or 10 mM H_2_O_2_ had higher SLA.

### Optimal photochemical efficiency of PSII

**Figure 7:**
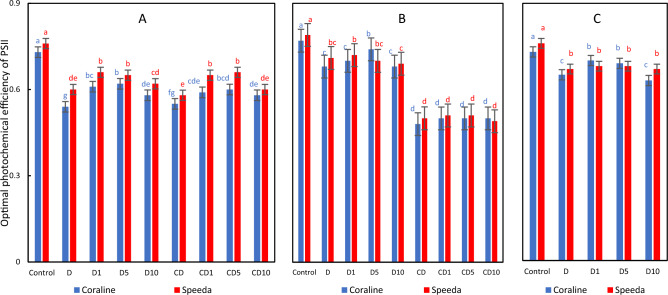
Optimal photochemical efficiency of PSII of two soybean genotypes (Coraline and Speeda) at 3 different sampling dates (**A**: 3 days after drought stress application at R1 stage, **B**: 3 days after R3 stage started, **C**: at the end of R4 stage) as affected by hydrogen peroxide foliar spray application under drought stress conditions (**D**: drought from R1 till R2 stage, D1: drought from R1 till R2 stage + 1 mM hydrogen peroxide, D5: drought from R1 till R2 stage + 5 mM hydrogen peroxide, D10: drought from R1 till R2 stage + 10 mM hydrogen peroxide, CD: continuous drought starting from R1 stage, CD1: continuous drought starting from R1 stage + 1 mM hydrogen peroxide, CD5: continuous drought starting from R1 stage + 5 mM hydrogen peroxide, CD10: continuous drought starting from R1 stage + 10 mM hydrogen peroxide). All values are the means of 3 replicates (columns) ± standard errors (vertical whiskers). In each genotype, different letters indicate significant differences at .05 level as indicated by Duncan’s multiple range test.

Drought stress caused a significant reduction in this trait after 3 days of application on both genotypes (by 26% and 21.1% in Coraline and Speeda, respectively). However, applying the foliar spray of all concentrations on Coraline and of 1 mM and 5 mM on Speeda led to significant enhancement in Fv/Fm. Compared to the treatments that were subjected to continuous drought, the group of treatments where the drought was relieved was able to maintain higher Fv/Fm values, with and without the application of H_2_O_2_ foliar spray and regardless of its concentration. Fv/Fm was significantly higher (by 8.8%) when 5 mM H_2_O_2_ foliar spray was applied to the recovering Coraline plants after 3 days of recovery compared to the non-sprayed recovering plants, whereas the foliar spray had no significant enhancement in Speeda recovering plants. Similar results were recorded at the end of the podding stage, where Fv/Fm values were not affected by the foliar spray on Speeda plants but were significantly better when 1 mM or 5 mM H_2_O_2_ was applied on Coraline plants (Fig. 7).

After drought application, Speeda plants had significantly higher Fv/Fm values in all treatments, except for the treatments where 10 mM H_2_O_2_ was applied (where the Fv/Fm values of Speeda was still higher, yet not significantly). However, there were no measurable differences between the two genotypes after the recovery process, i.e. Coraline plants were able to retain comparable Fv/Fm values when the plants had the chance to recover with and without foliar spray application (Fig. 7).

### Actual photochemical efficiency of PSII (Yield)

**Figure 8:**
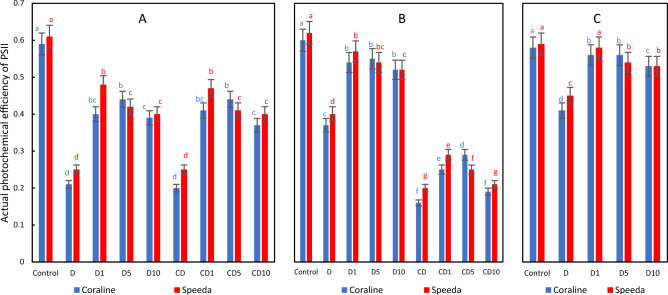
Actual photochemical efficiency of PSII of two soybean genotypes (Coraline and Speeda) at 3 different sampling dates (**A**: 3 days after drought stress application at R1 stage, **B**: 3 days after R3 stage started, **C**: at the end of R4 stage) as affected by hydrogen peroxide foliar spray application under drought stress conditions (**D**: drought from R1 till R2 stage, D1: drought from R1 till R2 stage + 1 mM hydrogen peroxide, D5: drought from R1 till R2 stage + 5 mM hydrogen peroxide, D10: drought from R1 till R2 stage + 10 mM hydrogen peroxide, CD: continuous drought starting from R1 stage, CD1: continuous drought starting from R1 stage + 1 mM hydrogen peroxide, CD5: continuous drought starting from R1 stage + 5 mM hydrogen peroxide, CD10: continuous drought starting from R1 stage + 10 mM hydrogen peroxide). All values are the means of 3 replicates (columns) ± standard errors (vertical whiskers). In each genotype, different letters indicate significant differences at .05 level as indicated by Duncan’s multiple range test.

The application of drought stress significantly reduced the actual photochemical efficiency of PSII of both genotypes 3 days after its application, regardless of H_2_O_2_ application and concentration. However, the exogenous application of H_2_O_2_ at any concentration significantly enhanced this trait in both genotypes as compared to the non-sprayed, drought-stressed treatment. After terminating the drought, the treatments that were sprayed with any concentration of H_2_O_2_ were still significantly higher in terms of yield as compared to the non-sprayed treatment. The group of treatments that were kept under drought stress conditions had significantly lower values of this trait. Within this group, the plants that received either 1 or 5, but not 10 mM H_2_O_2_, had significantly higher values than the treatment that did not receive any foliar H_2_O_2_. At the end of the podding stage, the actual photochemical efficiency of PSII of the treatments that received any concentration of H_2_O_2_ was still significantly higher than that of the treatment that did not receive H_2_O_2_ spray (Fig. 8).

### Chlorophyll-a

**Figure 9:**
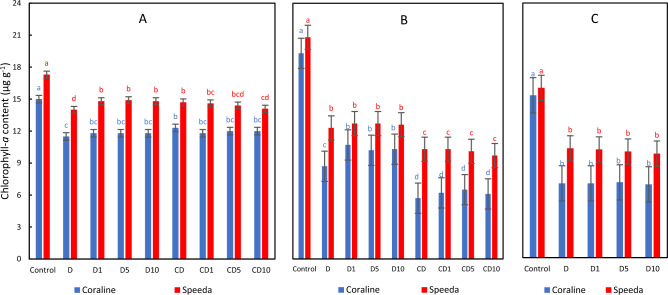
Chlorophyll-*a* content (µg g^-1^) of two soybean genotypes (Coraline and Speeda) at 3 different sampling dates (**A**: 3 days after drought stress application at R1 stage, **B**: 3 days after R3 stage started, **C**: at the end of R4 stage) as affected by hydrogen peroxide foliar spray application under drought stress conditions (**D**: drought from R1 till R2 stage, D1: drought from R1 till R2 stage + 1 mM hydrogen peroxide, D5: drought from R1 till R2 stage + 5 mM hydrogen peroxide, D10: drought from R1 till R2 stage + 10 mM hydrogen peroxide, CD: continuous drought starting from R1 stage, CD1: continuous drought starting from R1 stage + 1 mM hydrogen peroxide, CD5: continuous drought starting from R1 stage + 5 mM hydrogen peroxide, CD10: continuous drought starting from R1 stage + 10 mM hydrogen peroxide). All values are the means of 3 replicates (columns) ± standard errors (vertical whiskers). In each genotype, different letters indicate significant differences at .05 level as indicated by Duncan’s multiple range test.

After 3 days of drought stress application, the chlorophyll-*a* content significantly decreased in both genotypes, regardless of H_2_O_2_ application and concentration. In the drought-susceptible genotype Coraline, the treatments which were allowed to recover from drought stress had significantly higher chla content compared to the treatments which suffered from continuous drought stress. Among these treatments, the foliar application of H_2_O_2_, regardless of its concentration, led to significantly higher chla compared to the treatment where the plants were allowed to recover without H_2_O_2_ foliar spray. The foliar spray, however, did not have measurable effects on the chla content of the treatments which suffered from continuous drought. The foliar spray had no significant effect on the chla content in the drought-tolerant genotype Speeda; however, the group of treatments which was allowed to recover from drought stress had significantly higher chla content compared to the group which suffered from continuous drought. At the end of the podding stage, the control treatment had significantly higher chla content than the drought-stressed treatments (by 54% and 36% in Coraline and Speeda, respectively), and the foliar spray had no measurable effect (Fig. 9).

Under drought stress conditions, the chla content in Speeda was significantly higher than in Coraline at all 3 sampling dates, regardless of H_2_O_2_ application and concentration.

### Chlorophyll-b

**Figure 10:**
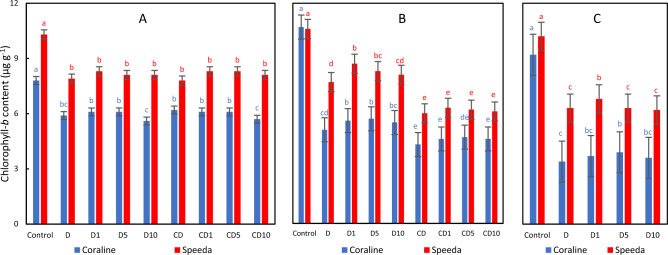
Chlorophyll-*b* content (µg g^-1^) of two soybean genotypes (Coraline and Speeda) at 3 different sampling dates (**A**: 3 days after drought stress application at R1 stage, **B**: 3 days after R3 stage started, **C**: at the end of R4 stage) as affected by hydrogen peroxide foliar spray application under drought stress conditions (**D**: drought from R1 till R2 stage, D1: drought from R1 till R2 stage + 1 mM hydrogen peroxide, D5: drought from R1 till R2 stage + 5 mM hydrogen peroxide, D10: drought from R1 till R2 stage + 10 mM hydrogen peroxide, CD: continuous drought starting from R1 stage, CD1: continuous drought starting from R1 stage + 1 mM hydrogen peroxide, CD5: continuous drought starting from R1 stage + 5 mM hydrogen peroxide, CD10: continuous drought starting from R1 stage + 10 mM hydrogen peroxide). All values are the means of 3 replicates (columns) ± standard errors (vertical whiskers). In each genotype, different letters indicate significant differences at .05 level as indicated by Duncan’s multiple range test.

The application of drought stress resulted in significant reduction in the chlb content in both genotypes. The foliar H_2_O_2_ spray could not alleviate that effect. However, the recovered plants of both genotypes had significantly higher content of chlb as compared to the plants where drought was continuous. Moreover, the application of H_2_O_2_ spray on the recovering plants of both genotypes increased the chlb content; that increase was significant when 1 mM or 5 mM of H_2_O_2_ foliar spray was applied. The foliar spray, on the other hand, did not result in significant enhancements in this trait under continuous drought stress conditions. At the end of the podding stage, the chlb content was significantly higher in Coraline (by 14.7%) and Speeda plants (by 7.9%) which were sprayed with 5 mM and 1 mM of H_2_O_2_, respectively compared to the non-sprayed counterparts (Fig. 10).

Speeda plants had significantly higher chlb content under drought stress conditions at all sampling dates.

### Total carotenoids

**Figure 11:**
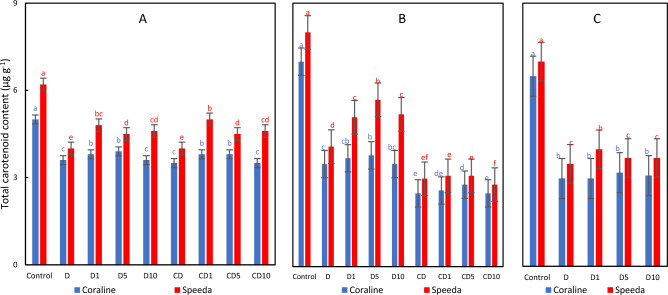
Total carotenoid content (µg g^-1^) of two soybean genotypes (Coraline and Speeda) at 3 different sampling dates (**A**: 3 days after drought stress application at R1 stage, **B**: 3 days after R3 stage started, **C**: at the end of R4 stage) as affected by hydrogen peroxide foliar spray application under drought stress conditions (**D**: drought from R1 till R2 stage, D1: drought from R1 till R2 stage + 1 mM hydrogen peroxide, D5: drought from R1 till R2 stage + 5 mM hydrogen peroxide, D10: drought from R1 till R2 stage + 10 mM hydrogen peroxide, CD: continuous drought starting from R1 stage, CD1: continuous drought starting from R1 stage + 1 mM hydrogen peroxide, CD5: continuous drought starting from R1 stage + 5 mM hydrogen peroxide, CD10: continuous drought starting from R1 stage + 10 mM hydrogen peroxide). All values are the means of 3 replicates (columns) ± standard errors (vertical whiskers). In each genotype, different letters indicate significant differences at .05 level as indicated by Duncan’s multiple range test.

Significant decrease in the total carotenoid (chlxc) content was recorded in both genotypes (by 28% and 35.5% in Coraline and Speeda, respectively) as a consequence of drought stress application. Except for the application of 10 mM H_2_O_2_ on Coraline plants, the chlxc content was significantly increased by the H_2_O_2_ foliar spray on both genotypes. The recovered plants of both genotypes had significantly higher chlxc content compared to the continuously drought-stressed counterparts. In Coraline plants, 1 mM H_2_O_2_ foliar spray on the recovering plants of Coraline significantly increased chlxc compared to the non-sprayed recovering plants, whereas the chlxc of recovering Speeda plants was significantly increased by the application of any concentration of H_2_O_2_. On the other hand, the foliar spray had no measurable effects on the chlxc of the plants that were not allowed to recover from both genotypes. Interestingly, the chlxc at the end of the podding stage was significantly higher in Speeda plants that were sprayed with 1 mM H_2_O_2_ as compared to the recovering plants that were not sprayed, whereas this trait did not have measurable differences in Coraline at the same period (Fig. 11).

Regardless of the drought application and the H_2_O_2_ application and concentration, the chlxc content was significantly higher in Speeda than in Coraline plants at all sampling dates.

### Stomatal conductance

**Figure 12:**
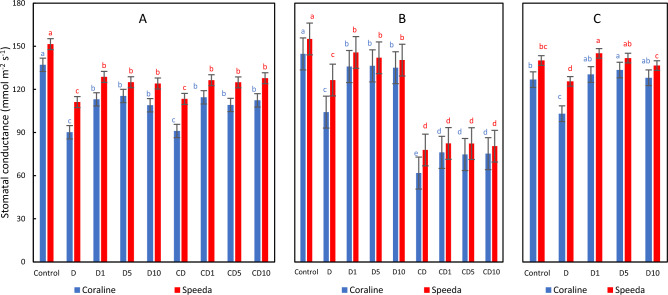
Stomatal conductance (mmol m^-2^ s^-1^) of two soybean genotypes (Coraline and Speeda) at 3 different sampling dates (**A**: 3 days after drought stress application at R1 stage, **B**: 3 days after R3 stage started, **C**: at the end of R4 stage) as affected by hydrogen peroxide foliar spray application under drought stress conditions (**D**: drought from R1 till R2 stage, D1: drought from R1 till R2 stage + 1 mM hydrogen peroxide, D5: drought from R1 till R2 stage + 5 mM hydrogen peroxide, D10: drought from R1 till R2 stage + 10 mM hydrogen peroxide, CD: continuous drought starting from R1 stage, CD1: continuous drought starting from R1 stage + 1 mM hydrogen peroxide, CD5: continuous drought starting from R1 stage + 5 mM hydrogen peroxide, CD10: continuous drought starting from R1 stage + 10 mM hydrogen peroxide). All values are the means of 3 replicates (columns) ± standard errors (vertical whiskers). In each genotype, different letters indicate significant differences at .05 level as indicated by Duncan’s multiple range test.

The stomatal conductance significantly decreased in both genotypes when subjected to drought stress. The foliar application of H_2_O_2_ at any concentration could significantly elevate the stomatal conductance of both Coraline and Speeda plants. The stomatal conductance of both genotypes dramatically degraded when drought stress was kept; however, the foliar spray with H_2_O_2_ at all concentrations could significantly increase the stomatal conductance of Coraline (by an average 22%), but not that of Speeda plants. On the other hand, the recovered plants of both genotypes had significantly higher stomatal conductance when foliar spray was applied at any concentration as compared to the recovering plants that was not sprayed. Interestingly, Coraline and Speeda plants that were sprayed with 5 mM and 1 mM H_2_O_2_, respectively had significantly higher stomatal conductance values (by 5.3% and 3.7%) compared to the control counterparts that were kept under optimum conditions throughout the whole experimental period (Fig. 12).

Speeda plants could maintain higher stomatal throughout the experimental period as compared to Coraline plants.

### Relative water content

The relative water content of all drought-stressed treatments of both genotypes significantly decreased as compared to control treatment, regardless of H_2_O_2_ application and concentration. On the other hand, the exogenous application of either 1 or 5 mM H_2_O_2_ on Coraline, and 1 mM H_2_O_2_ on Speeda significantly enhanced the RWC. After terminating drought, a very similar result was obtained, where these concentrations helped in elevating the RWC of both genotypes to reach nearly similar values to those of control plants. On the other hand, all the treatments where the plants of both genotypes were kept under drought stress conditions had significantly lower RWC, yet all treatments that received any concentration of H_2_O_2_ (except for 10 mM on Speeda) were able to keep significantly better RWC as compared to the non-sprayed counterpart. At the end of the podding stage, the RWC of (D1) and (D5) treatments in Coraline, and of (D1) treatment in Speeda was significantly higher than that of (D) treatment (Table 1).

**Table 1:** Relative water content (%) of two soybean genotypes (Coraline and Speeda) at 3 different sampling dates as affected by hydrogen peroxide foliar spray application under drought stress conditions (D: drought from R1 till R2 stage, D1: drought from R1 till R2 stage + 1 mM hydrogen peroxide, D5: drought from R1 till R2 stage + 5 mM hydrogen peroxide, D10: drought from R1 till R2 stage + 10 mM hydrogen peroxide, CD: continuous drought starting from R1 stage, CD1: continuous drought starting from R1 stage + 1 mM hydrogen peroxide, CD5: continuous drought starting from R1 stage + 5 mM hydrogen peroxide, CD10: continuous drought starting from R1 stage + 10 mM hydrogen peroxide).

Sampling date	3 days after drought stress application at R1 stage		3 days after R3 stage started		At the end of R4 stage	
Treatment	Coraline	Speeda	Coraline	Speeda	Coraline	Speeda
Control	70.5^a^	72^a^	68.9^a^	71^a^	66.6^a^	67.4^ab^
D	52.4^d^	59.1^c^	58.4^b^	64.9^b^	57.6^c^	62.1^b^
D1	59.4^c^	65.6^b^	64.2^a^	70.1^a^	62.2^b^	68.7^a^
D5	64.8^b^	63.7^bc^	65^a^	65.2^b^	63.5^a^	64^b^
D10	54.1^d^	62.9^bc^	62.3^b^	65^b^	60^bc^	62.9^b^
CD	52^d^	59.2^c^	40.2^d^	49.3^d^	NA	NA
CD1	59.5^c^	65.3^b^	47.6^c^	57.7^c^	NA	NA
CD5	65^b^	63.6^bc^	49.1^c^	54.8^c^	NA	NA
CD10	54.2^d^	63^bc^	47.5^c^	53.9^ cd^	NA	NA

All values are the means of 3 replicates. In each genotype, different letters indicate significant differences at .05 level as indicated by Duncan’s multiple range test. NA: Not Applicable.

At all sampling dates, the RWC of Speeda was significantly higher than that of Coraline in all drought treatments except for the D5 treatment.

### Flower number

At the end of the flowering stage, the treatments of both genotypes that were subjected to drought stress without any foliar spray produced significantly lower number of flowers (by 19.7% and 26.9% in Coraline and Speeda, respectively) as compared to control counterparts. The foliar spray enhanced this trait in Coraline, where both 1 mM and 5 mM H_2_O_2_ concentrations resulted in significantly higher flower number as compared to the non-sprayed treatments. However, the foliar spray did not enhance this trait in Speeda; it even decreased the flower number when 5 mM or 10 mM H_2_O_2_ was applied (Table 2).

**Table 2:** Flower number of two soybean genotypes (Coraline and Speeda) at full bloom (R2) stage as affected by hydrogen peroxide foliar spray application under drought stress conditions (D: drought from R1 till R2 stage, D1: drought from R1 till R2 stage + 1 mM hydrogen peroxide, D5: drought from R1 till R2 stage + 5 mM hydrogen peroxide, D10: drought from R1 till R2 stage + 10 mM hydrogen peroxide, CD: continuous drought starting from R1 stage, CD1: continuous drought starting from R1 stage + 1 mM hydrogen peroxide, CD5: continuous drought starting from R1 stage + 5 mM hydrogen peroxide, CD10: continuous drought starting from R1 stage + 10 mM hydrogen peroxide).

Treatment	Coraline	Speeda
Control	18.3^a^	28.3^a^
D	14.7^c^	20.7^b^
D1	16.3^b^	21.3^b^
D5	18^a^	17.7^c^
D10	14.7^c^	17^c^
CD	14.6^c^	20.9^b^
CD1	16.4^b^	21.2^b^
CD5	17.9^a^	17.6^c^
CD10	14.5^c^	17.1^c^

All values are the means of 3 replicates. In each genotype, different letters indicate significant differences at .05 level as indicated by Duncan’s multiple range test.

Speeda had significantly higher flower number than Coraline in all treatments except for the treatments that were sprayed with 5 mM H_2_O_2_ where the flower numbers were very similar.

### Pod number

The number of pods of both genotypes decreased due to drought stress application; the reduction was more measurable and significant in Speeda (37%). The pod number significantly increased in Coraline when 1 mM or 5 mM H_2_O_2_ foliar spray was applied, and the same result was obtained when 1 mM H_2_O_2_ foliar spray was applied on Speeda (Table 3).

**Table 3:** Pod number (plant^-1^) of two soybean genotypes (Coraline and Speeda) at the end of R4 stage as affected by hydrogen peroxide foliar spray application under drought stress conditions (D: drought from R1 till R2 stage, D1: drought from R1 till R2 stage + 1 mM hydrogen peroxide, D5: drought from R1 till R2 stage + 5 mM hydrogen peroxide, D10: drought from R1 till R2 stage + 10 mM hydrogen peroxide.

Treatment	Coraline	Speeda
Control	15.3^a^	22.7^a^
D	11.3^b^	14.3^c^
D1	14^a^	17.7^b^
D5	13.6^a^	13.7^c^
D10	8.3^c^	9.7^d^

All values are the means of 3 replicates. In each genotype, different letters indicate significant differences at .05 level as indicated by Duncan’s multiple range test.

The number of pods was higher for Speeda in all treatments as compared to Coraline.

### Pod fresh weight

Significant decrease in the pod fresh weight under drought stress conditions was recorded in both genotypes. However, the application of 5 mM and 1 mM H_2_O_2_ foliar spray on Coraline and Speeda, respectively has led to significant increase (by 18.1% and 14.1%, respectively) in the pod fresh weight (Table 4).

**Table 4:** Pod fresh weight (g plant^-1^) of two soybean genotypes (Coraline and Speeda) at the end of R4 stage as affected by hydrogen peroxide foliar spray application under drought stress conditions (D: drought from R1 till R2 stage, D1: drought from R1 till R2 stage + 1 mM hydrogen peroxide, D5: drought from R1 till R2 stage + 5 mM hydrogen peroxide, D10: drought from R1 till R2 stage + 10 mM hydrogen peroxide.

Treatment	Coraline	Speeda
Control	19.3^a^	34.7^a^
D	12.7^c^	21.3^c^
D1	14.7^bc^	24.3^b^
D5	15^b^	17^d^
D10	12.7^c^	14.7^e^

All values are the means of 3 replicates. In each genotype, different letters indicate significant differences at .05 level as indicated by Duncan’s multiple range test.

The pod fresh weight of Speeda was significantly higher than that of Coraline, regardless of drought and H_2_O_2_ foliar spray application.

### Proline content

The leaf proline content of both genotypes significantly increased under drought stress conditions. Furthermore, the foliar application of H_2_O_2_ at any concentration significantly increased the leaf proline content as compared to the treatment where the drought-stressed plants were sprayed with DW. When drought was terminated, proline content measurably decreased in both genotypes, yet it was still higher than that of control treatment. Under continuous drought conditions, proline continued to accumulate, and its levels were significantly higher than those of the drought-relieved counterparts. The foliar H_2_O_2_ spray had no measurable effect at this point in any of the two genotypes. The leaf proline content was still higher in the drought-relieved treatments at the end of the podding stage as compared to the control counterparts (Table 5).

**Table 5:** Leaf proline content (µg g^-1^) of two soybean genotypes (Coraline and Speeda) at 3 different sampling dates as affected by hydrogen peroxide foliar spray application under drought stress conditions (D: drought from R1 till R2 stage, D1: drought from R1 till R2 stage + 1 mM hydrogen peroxide, D5: drought from R1 till R2 stage + 5 mM hydrogen peroxide, D10: drought from R1 till R2 stage + 10 mM hydrogen peroxide, CD: continuous drought starting from R1 stage, CD1: continuous drought starting from R1 stage + 1 mM hydrogen peroxide, CD5: continuous drought starting from R1 stage + 5 mM hydrogen peroxide, CD10: continuous drought starting from R1 stage + 10 mM hydrogen peroxide).

Sampling date	3 days after drought stress application at R1 stage		3 days after R3 stage started		At the end of R4 stage	
Treatment	Coraline	Speeda	Coraline	Speeda	Coraline	Speeda
Control	30.7^d^	33^c^	43.4^c^	47.3^c^	39.3^b^	42^b^
D	226.4^c^	272.6^b^	183.4^b^	240.3^b^	92.7^a^	112.9^a^
D1	253.9^b^	391.7^a^	142.9^b^	166.7^b^	81.1^a^	100.5^a^
D5	280^a^	379.3^a^	127.6^b^	186.2^b^	74.6^a^	103.3^a^
D10	275.8^ab^	365.4^a^	130^b^	180.8^b^	79.9^a^	101^a^
CD	228.3^c^	270.6^b^	402.7^a^	468.1^a^	NA	NA
CD1	255.1^b^	390.6^a^	449.5^a^	521.7^a^	NA	NA
CD5	278.4^a^	376^a^	472.8^a^	485.5^a^	NA	NA
CD10	275^ab^	365.3^a^	411.4^a^	447^a^	NA	NA

All values are the means of 3 replicates. In each genotype, different letters indicate significant differences at .05 level as indicated by Duncan’s multiple range test. NA: Not Applicable.

The leaf proline content was always higher in Speeda than in Coraline, and the differences were significant in all drought-stressed treatments of both groups.

### Total soluble sugars

Drought stress significantly increased the total soluble sugars in the leaves of both genotypes. Compared to the drought-stressed treatment, the application of H_2_O_2_ foliar spray at any concentration in Coraline, and at 1 or 5 mM in Speeda significantly induced the accumulation of soluble sugars. When drought was eliminated after the flowering stage, the total soluble sugars in Coraline noticeably decreased and reached levels that were insignificant as compared to the control plants, whereas these levels were still significantly higher in Speeda. Furthermore, the sprayed plants of both genotypes had very close levels of soluble sugars as compared to the non-sprayed counterparts. On the other hand, the group of treatments that had continuous drought accumulated significant levels of soluble sugars as compared to the drought-relieved group of both genotypes. In Coraline, the soluble sugar contents were not significantly different in the treatments sprayed with any concentration of H_2_O_2_ from the treatment that was not sprayed, whereas they were in Speeda. At the end of the podding stage, the content of the total soluble sugars was still significantly higher in the treatments that suffered from drought, regardless of H_2_O_2_ application and concentration, as compared to the control treatments that were sprayed with DW (Table 6).

**Table 6:** Total soluble sugars (mg g^-1^) in the leaves of two soybean genotypes (Coraline and Speeda) at 3 different sampling dates as affected by hydrogen peroxide foliar spray application under drought stress conditions (D: drought from R1 till R2 stage, D1: drought from R1 till R2 stage + 1 mM hydrogen peroxide, D5: drought from R1 till R2 stage + 5 mM hydrogen peroxide, D10: drought from R1 till R2 stage + 10 mM hydrogen peroxide, CD: continuous drought starting from R1 stage, CD1: continuous drought starting from R1 stage + 1 mM hydrogen peroxide, CD5: continuous drought starting from R1 stage + 5 mM hydrogen peroxide, CD10: continuous drought starting from R1 stage + 10 mM hydrogen peroxide).

Sampling date	3 days after drought stress application at R1 stage		3 days after R3 stage started		At the end of R4 stage	
Treatment	Coraline	Speeda	Coraline	Speeda	Coraline	Speeda
Control	42.5^c^	45.9^d^	36.1^b^	40.2^d^	33.3^b^	35.8^b^
D	123.8^b^	165.2^c^	65^b^	84.4^c^	51.3^a^	59.7^a^
D1	160.1^a^	228.3^a^	68.9^b^	90.4^c^	53.9^a^	61.1^a^
D5	182.7^a^	215.8^ab^	70.9^b^	86.1^c^	54.1^a^	60.8^a^
D10	173.9^a^	195^bc^	69^b^	84.9^c^	52.8^a^	59.7^a^
CD	125^b^	163.9^c^	175.3^a^	235.7^b^	NA	NA
CD1	162.1^a^	230.4^a^	184.9^a^	286.4^a^	NA	NA
CD5	177.7^a^	213.8^ab^	189.2^a^	271.9^a^	NA	NA
CD10	173.5^a^	195.5^bc^	186.6^a^	262.8^a^	NA	NA

All values are the means of 3 replicates. In each genotype, different letters indicate significant differences at .05 level as indicated by Duncan’s multiple range test. NA: Not Applicable.

Although Speeda plants had higher contents of the total soluble sugars, yet the differences were more announced after drought stress application, whereas these differences were much less in the group of treatments that was relieved from drought, and also at the end of the podding stage.

## Discussion

Osmotic stress can limit energy transport from photosystem II to photosystem I, and parallelly constitute spongy, thin tissues in the leaves, leading to elevated chlorophyll-*a* fluorescence and, consequently, reduced photosynthetic activity^52^. ROS accumulation negatively affects the sensitive chlorophyll molecules^53^. In our experiment, both chlorophyll-*a* and chlorophyll-*b* of both soybean genotypes significantly decreased under PEG-induced drought stress conditions, leading to damaged photosynthesis machinery^54^. Similar conclusion was reported by^55^, who also reported that total carotenoids significantly decreased under drought stress conditions, which was the case in our experiment as well. The exogenous application of either 1 or 5 mM H_2_O_2_ could measurably enhance chlorophyll-*a* content in the drought-tolerant genotype Speeda, but not of the drought-susceptible genotype Coraline. However, no influence on Chl b content in both genotypes was detected. On the other hand, the total carotenoid content in both genotypes was significantly enhanced by H_2_O_2_ application at any concentration (except for 10 mM on Coraline). Low concentrations of H_2_O_2_ can induce certain enzymes and/or proteins related to photosynthesis process^56^. H_2_O_2_ foliar spray can protect the chloroplast under drought stress conditions, resulting in enhanced chlorophyll content^37,57^. Similar conclusions were also reported on soybean in the case of exogenous melatonin^58^ and ethanol^59^. Ethanol application can elevate the synthesis and/or reduce the degradation of the photosynthetic pigments^55^.

The drought-stressed plants of both soybean genotypes in our experiment had significantly higher proline and soluble sugar concentrations 3 days after drought stress application. Proline is an important amino acid that is engaged in many processes on the cellular level^60^. Under drought stress conditions, the concentrations of proline and soluble sugars, among other osmolytes, increase without disturbing the usual biochemical activities in the cells^61^. Thus, these osmolytes play a defensive role against drought by decreasing the permeability of the cellular membranes, leading to stabilized water balance^62–68^. In their experiment^58^, reported that drought-stressed soybean seedlings had 30, 125 and 334% higher proline concentration after 5, 10 and 15 days of drought stress application. Such conclusion on proline and soluble sugar accumulation was also reported on soybean by other studies (e.g.^69–71^) and on other species like hot pepper^72^, barley^73^, cotton^74^ and rice^75^. According to^76^, there is another important role of the elevated soluble sugar levels under drought stress conditions; that is, sustaining adequate metabolic C/N ratios. The concentrations of both proline and soluble sugars were measurably higher in Speeda than in Coraline at the 3 sampling dates. It was previously reported that the levels of proline accumulations are genotype-dependent and varies among the different stages of soybean development when the drought stress is taking place^12,77^, which is also confirmed by our findings, as the group of treatments of both genotypes that was relieved from drought stress after flowering stage had significantly higher concentrations of both proline and soluble sugars as compared to the other group, where the drought was continuously kept in place. On the other hand, the exogenous application of H_2_O_2_ noticeably increased both proline and soluble sugar concentrations in the drought-stressed plants of both genotypes 3 days after drought stress application. It was reported by^36^ that H_2_O_2_ foliar spray resulted in elevated proline and soluble sugar concentrations in drought-stressed maize plants, leading to enhanced drought tolerance. Similar conclusion was also reported when other osmo-regulators such as ethanol^55^ were exogenously applied on soybean plants.

Under unfavorable water availability conditions, sustaining water status within plants is vital to overcome these conditions, and leaf relative water content is considered as one of the most indicative traits for plant drought tolerance^78^. In our experiment, significant reduction in RWC under drought stress conditions was recorded in both soybean genotypes. It is well documented that drought stress results in reduced stomatal conductance by increasing stomatal closure ratio^79^ in order to maintain the water content of the drought-stressed plants. In their experiment^80^, concluded that RWC of both experimented soybean genotypes decreased under drought stress conditions, and^55,81^ reported that gs significantly decreased in drought-stressed soybean plants, which was also supported by our results. However, Significant enhancement in gs was recorded when any concentration of H_2_O_2_ was exogenously applied on both soybean genotypes. Simultaneously, the RWC was significantly better 3 days after drought stress imposition when 1 or 5 mM H_2_O_2_ was applied on Coraline plants and 1 mM H_2_O_2_ was applied on Speeda plants. Previously^35,36,82^. reported significant enhancement in both RWC and gs in drought-stressed soybean as a consequence of H_2_O_2_ foliar application. This conclusion was also reported when drought-stressed soybean plants were exogenously treated with melatonin, and when rice plants were exogenously sprayed with either H_2_O_2_ or SA^58,75^.

Both the optimal (Fv/Fm) and the actual photochemical efficiency of PSII (yield) significantly decreased in both genotypes 3 days after drought stress imposition. However, the exogenous application of any concentration of H_2_O_2_ significantly enhanced these traits in both genotypes (except for the Fv/Fm of Speeda plants sprayed with 10 mM H_2_O_2_). It was previously reported that a key factor of plant’s response to variations in the surrounding environment is the chlorophyll fluorescence^83^. Under drought stress conditions, leaves absorb and translocate less energy to PSII, leading to inhibited plant development^84^. Similar to our findings^81^, reported that Fv/Fm significantly decreased under PEG-induced drought stress.

The capacity of light intercepted by plants, and thus of photosynthetic rate is measurably affected by the plant’s leaf area^85,86^. It is well documented that drought stress results in reduced leaf area (e.g.^87,88^). Our results also are in agreement with this conclusion. Furthermore, we found out that plants of both genotypes that were sprayed with any concentration of H_2_O_2_ could noticeably maintain higher SLA at all 3 sampling dates. It was also found out earlier that the exogenous spray of H_2_O_2_^89^, ethanol^55^ and SA^90,91^ could enhance the leaf area of soybean plants.

Drought stress can alter plant’s growth and development through several changes in the root^92^ and shoot^89^ levels. We found out that the root DW, root volume and root length increased 3 days after drought stress imposition as compared to control plants of both genotypes, with more announced increase in Coraline, the drought-susceptible genotype. Increased root length under drought stress conditions can help the plants acquire more water and nutrients from deeper soil levels^93,94^. On the other hand, shoot length and DW noticeably decreased under drought stress conditions; similar conclusion was also reported by^95^. The application of H_2_O_2_ enhanced both root and shoot length of Speeda, but not Coraline, whereas did not enhance the DW^96^ concluded that H_2_O_2_ enhances root growth by increasing ABA levels. It was previously reported that the roots and the seedlings of sweet potato responded positively to the application of H_2_O_2_ at levels between 0.5 and 2.5 mM, but negatively at levels of 5 mM or more^97^.

## Conclusions

Drought stress affecting soybeans during flowering stages impairs several important morpho-physiological traits, leading to massive losses in reproductive organs and, consequently, potential yields. A post-drought recovery can still enable the plants survive and reach maturity stages. However, the continuous severe drought occurring for longer periods during reproductive stages can lead to even complete deterioration of plants. The exogenous application of H_2_O_2_ in low concentrations can help soybean plants overcome most of these impairments and enhance the root morphology, enabling for more water and nutrient uptake potentials under this unfavorable condition. This treatment was more effective on the drought-susceptible than on the drought-tolerant soybean genotype, with more announced enhancements of the stomatal conductance, relative water content, specific leaf area, root and shoot lengths and leaf proline and soluble sugar concentrations, where the plants had eventually similar levels of these traits as compared to the control treatments. A higher concentration of 10 mM H_2_O_2_ had little to no effect on the majority of the studied traits.

## Data Availability

All data generated or analyzed during this study are included in this published article.
